# Response of Tomato Plants, *Ailsa Craig* and Carotenoid Mutant *tangerine*, to Simultaneous Treatment by Low Light and Low Temperature

**DOI:** 10.3390/plants13141929

**Published:** 2024-07-12

**Authors:** Antoaneta V. Popova, Martin Stefanov, Gergana Mihailova, Preslava Borisova, Katya Georgieva

**Affiliations:** 1 Institute of Biophysics and Biomedical Engineering, Bulgarian Academy of Sciences, Acad, G. Bonchev Str. Bl. 21, 1113 Sofia, Bulgaria; martin@bio21.bas.bg (M.S.); preslavab12345@gmail.com (P.B.); 2 Institute of Plant Physiology and Genetics, Bulgarian Academy of Sciences, Acad, G. Bonchev Str. Bl. 21, 1113 Sofia, Bulgaria; gmihailova@bio21.bas.bg (G.M.); georgieva.katya.m@gmail.com (K.G.)

**Keywords:** *Solanum lycopersicum*, carotenoid mutant *tangerine*, low light, low temperature, abiotic stress, antioxidants

## Abstract

Tomato (*Solanum lycopersicum* L.) plants, wild type *Ailsa Craig*, and carotenoid mutant *tangerine* that accumulates prolycopene instead of all-*trans*-lycopene were exposed to a combined treatment by low light and low temperature for 5 days. The ability of plants to recover from the stress after development for 3 days at control conditions was followed as well. The suffered oxidative stress was evaluated by the extent of pigment content, lipid peroxidation, membrane stability, and H_2_O_2_ generation. The level of MDA content under combined treatment in *tangerine* implies that the mutant demonstrates lower sensitivity to stress in comparison with *Ailsa Craig*. The oxidative protective strategy of plants was estimated by following the antioxidant and antiradical activity of phenolic metabolites, including anthocyanins, as well as the activities of antioxidant enzymes superoxide dismutase (SOD), ascorbate peroxidase (APX) and catalase (CAT). Presented results revealed that the oxidative stress was much stronger expressed after exposure of both types of plants to low light combined with low temperature compared to that after treatment with only low light. The most significant antioxidant protection was provided by phenolic substances, including anthocyanins. The lower sensitivity of *tangerine* plants to low light can be attributed to the higher activity of the antioxidant enzyme CAT.

## 1. Introduction

The ever-changing environmental conditions seriously affect the development and distribution of higher plants. The spectral characteristics and intensity of light as well as deviation from the respective optimal temperature, are the most important environmental factors that impact in a specific manner every genotype [1]. Light quality and quantity strongly affect photosynthetic processes, carbon metabolism, and plant growth-related parameters at physiological, biochemical, and molecular levels. Receiving less light than required for the proper development suppresses growth and metabolic reactions and causes significant alterations in plant morphology—an increase in plant height, leaf number, and specific leaf area aiming to absorb as much light as possible to ensure the effectivity of photosynthetic reactions [2,3]. At low light intensities plants show lower photosynthetic performance and less dry matter accumulation and are more susceptible to photoinhibition in comparison with those grown at high light intensities [3]. Low-light tolerant species can maintain more efficient photosynthetic rates due to maintenance of high chlorophyll content, enhanced antioxidant enzyme activities, osmotic adjustments, and lower level of lipid peroxidation in comparison with plants that cannot tolerate development at low light availability [3].

Another limiting factor for plant development and productivity is low temperature. Exposure to lower than optimal temperature has a negative effect on water availability; the physicochemical properties of the lipid bilayer of biological membranes are altered, and an imbalance between absorbed excitation energy and metabolic sinks is observed as the rate of enzymatic reactions is restricted at lower than optimal temperature [1,4,5,6]. To cope with challenging conditions of development at low temperatures, plants increase the accumulation of sugars to prevent dehydration, synthesis of anthocyanins and antioxidant enzymes is activated to mitigate the severity of oxidative stress caused by extreme environmental deviation [6].

The simultaneous treatment by low light and low temperature seriously restricts photosynthetic efficiency, biological carbon, and nitrogen fixation, significantly increases the generation of reactive oxygen species (ROS), and consequently elevates membrane lipid peroxidation [7,8]. Plants exposed to a combined low temperature and weak light stress have thinner and larger leaves compared with the leaves of plants grown under normal growth conditions [8,9]. The response of plants to the simultaneous application of these two stress factors is determined by a complex interaction between light- and temperature-mediated signaling processes expressed in the induction of stress-related genes and synthesis of protective metabolites [10]. Induction of genes involved in antioxidant protection against low light in Arabidopsis occurs at the availability of sufficient light [11]. Furthermore, cold acclimation of plants is more effective when exposed to low but not freezing conditions in the presence of normal or high light than in dark or low light intensities [10]. In addition, induction of some antioxidant enzymes (glutathione reductase and ascorbate peroxidase) is observed in wheat only under normal growth conditions but not at reduced light intensity [12]. Accumulation of stress-protective substances, including polyamines, proline, phenolic substances, etc., is also induced in a light-dependent manner in cold hardened wheat plants [12,13].

In photosynthetic organisms the electron transport system of chloroplasts generates ROS even under optimal conditions that are significantly accelerated when plants are suffering abiotic stress [14,15]. The main types of ROS are superoxide anion (O2^−^), hydrogen peroxide (H_2_O_2_), singlet oxygen (^1^O_2_), and hydroxyl radicals (OH^−^) [14,15,16]. Singlet oxygen is generated in the super complex of photosystem II (PSII) after energy exchange between the triplet excited state of chlorophyll in the reaction center and O_2_ (^3^P680*) [17] while O_2_^−^ is generated at the acceptor site of PSI. The generated ROS are extremely harmful to cell components as they can oxidize lipids, proteins, and DNA. Thylakoid membranes, characterized by a high degree of unsaturation of their fatty acid chains, are very vulnerable to oxidative stress [18].

Plants have developed different strategies to alleviate the detrimental effect of oxidative stress, including increased accumulation of antioxidant compounds as ascorbate, tocopherols, and phenolic substances, as well as enhanced activity of antioxidant enzymes such as superoxide dismutase (SOD), ascorbate peroxidase (APX) and catalase (CAT) [15,16].

Phenolic compounds represent a substantial group of secondary metabolites acting as primary antioxidants and free radical scavengers, estimated by FRAP and DPPH assays, respectively, protecting plants from oxidative stress. Due to the aromatic ring, phenols are able to stabilize and relocate the unpaired electrons of their structure, facilitating the donation of hydrogen atoms and electrons from their hydroxyl groups [19].

Anthocyanins are a diverse group of secondary metabolites that belong to the flavonoid family. Accumulation of anthocyanins is related to various types of abiotic stress conditions, especially to high light illumination. Anthocyanins are located in plant cell vacuoles and their main function is to screen the excessively absorbed light. However, some anthocyanins have been shown to possess much higher antioxidant activity in comparison with well-established antioxidants such as ascorbate and tocopherol [20,21,22].

The antioxidant enzyme SOD is involved in the dismutation of O_2_^−^ to H_2_O_2_ and O_2_, thus providing protection against damaging oxidative action of O_2_^−^ [14,15,23,24]. The dangerous H_2_O_2_ is relatively stable and can alter the redox state of different cell organelles and molecules but additionally can serve as an abiotic stress signaling molecule in plants [16]. The enzymes APX and CAT provide protection against oxidative stress by scavenging generated H_2_O_2_ [24,25,26].

Many horticultural crops, such as cotton, maize, rice, tobacco, tomato, etc., originate from tropical/subtropical regions and require high light supply and high temperatures for development and productivity [27]. Development at suboptimal light and temperature conditions seriously reduces biomass production and yield of tomato [28,29,30], sweet pepper [31], rice [3], and maize [32]. Tomato (*Solanum lycopersicum* L.) is one of the most important agricultural vegetable crops, used as a source of carotenoids (lycopene) for humans. It is cultivated under controlled conditions during winter and spring to meet the increasing demands of a healthy human diet. Due to its tropical origin development of tomato plants at reduced light supply and suboptimal temperatures seriously reduces plants’ growth and production of fruits [29]. However, reported data about the combined effects of low temperature and low light on the development and productivity of tomato plants are rather limited.

Photosynthetic pigments, chlorophylls, and carotenoids are intrinsic components of photosynthetic apparatus responsible for sunlight absorption, transfer, and conversion of solar energy into electrochemical energy. The development of chloroplasts and synthesis of chlorophyll in young plant leaves are negatively influenced by low-light treatment [33]. However, the response of plants to treatment by low light intensity is dependent on the tolerance of the respective genotype to low light, duration of treatment, developmental stage, etc. Low light-tolerant plants increase the content of chlorophyll b and decrease the Chl a/b ratio, indicating increased light-harvesting antenna size that enables the plants to absorb as much light as possible, even at reduced light availability [3].

Carotenoids perform multiple functions, including light harvesting, antioxidant, photoprotective, and structural functions [33]. The preferred conformation of carotenoids in plants is all-*trans*-isomer, but *cis*-isomers are also available, however, in a much lower quantity [34]. The majority of published data concerning carotenoid content discuss the role of carotenoids in the formation of fruits in crops [35,36,37,38]. Information about the effect of carotenoid content on the response of plants to low light and low light in combination with low temperature is rather limited. A significant increase of zeaxanthin was detected in pepper (*Capsicum annuum* L.) seedlings after exposure to low light and low temperature, while treatment only by low light led to elevated content of β-carotene [39]. The availability of mutants with altered carotenoid content or ratio between carotenoid species facilitates the unraveling of the contribution of different carotenoid species to the response of higher plants to extreme environmental conditions. In this study we used the *tangerine* mutant of tomato (*Solanum lycopersicum*) that is characterized by a mutation in the CrtISO gene [40]. The *tangerine* mutant contains defective prolycopene isomerase (CRTISO) that is performing the isomerization of tetra-*cis*-lycopene to all-*trans*-lycopene [38,40] and tomato plants accumulate prolycopene instead of all-*trans*-lycopene. The fruits of *tangerine* are orange in color, young leaves are yellowish, and flowers are pale.

The aim of the present report was to evaluate the response of tomato plants, wild type *Ailsa Craig*, and carotenoid mutant *tangerine* (locus t) to short time (5 days) exposure of young plants to simultaneous treatment by low light intensity (125 µmol photons m^−2^ s^−1^) and suboptimal (15/10 °C day/night) temperature. The ability of plants to recover from the stress in 3 days under control conditions was followed as well. We applied low light treatment as the light and chlorophyll can substitute the role of the defective prolycopene isomerase (CRTISO), as was suggested by Isaacson et al. [40] and Enfissi et al. [37]. The extent of suffered stress by *Ailsa Craig* and *tangerine* plants was monitored by the level of electrolyte leakage, generation of H_2_O_2_, photosynthetic pigment content, and degree of lipid peroxidation. The protective strategies of tomato plants were evaluated by the antioxidant and antiradical activity of phenolic compounds, accumulation of anthocyanins, and the activities of antioxidant enzymes (SOD, APX and CAT). The investigation was performed on leaves of control, treated, and recovered plants.

## 2. Results

**Figure 1:**
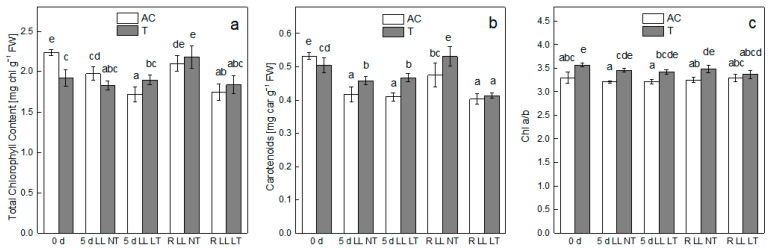
Alterations in photosynthetic pigment content—total chlorophyll content—Chl (a+b) (**a**), carotenoids (**b**) and Chl a/b ratio (**c**) in *Ailsa Craig* (AC) and carotenoid mutant *tangerine* (T) as induced by treatment of whole plants for 5 days by low light illumination (LL) in combination with normal (NT) or low (LT) temperature and after recovery period of 3 days (R). Data are presented as [mg pigment g^−1^ FW]. Mean values ± SE were calculated from two independent experiments and four parallel samples at each time point (*n* = 8). Significant differences between values at *p* < 0.05, as estimated by Fisher’s LSD test of ANOVA, were indicated with different letters.

The effect of treatment by low light (LL) in combination with control (NT) or low (LT) temperature on the pigment content in leaves of *Ailsa Craig* and *tangerine* plants was presented in (Figure 1). At control growth conditions, *Ailsa Craig* plants showed higher pigment content in comparison with *tangerine*. In non-treated mutant plants, the total chlorophyll content (Chl (a+b)) was lower by 15%, being 1.919 ± 0.103 (mg Chl (a+b) g^−1^ FW) in comparison with 2.238 ± 0.038 (mg Chl (a+b) g^−1^ FW) for *Ailsa Craig*. The total chlorophyll content in *Ailsa Craig* leaves decreased after 5 days of treatment by LL NT by 12% and by 23% when LL was combined with LT. However, in *tangerine* leaves, the chlorophyll content was slightly decreased after both treatments but the values were not statistically different in comparison with control plants. Transfer of *Ailsa Craig* and *tangerine* plants to control conditions led to the recovery of chlorophyll content in plants treated by LL NT, while both types of plants that were exposed to LL LT showed chlorophyll content comparable with that of treated ones (Figure 1a). Similar to chlorophyll content the level of carotenoids in control of mutant plants was lower in comparison with *Ailsa Craig*, but by only around 5%. Exposure to both types of treatment, LL NT and LL LT, led to a comparable decline in carotenoid content by 22% for *Ailsa Craig* and, to a lesser extent, for *tangerine* plants—by 10%. After the transfer of plants to control conditions, recovery of carotenoid content was detected after treatment by LL NT. The recovery process was not so effective after exposure to LL LT. *Ailsa Craig* plants showed carotenoid content similar to that of treated ones, while *tangerine* plants demonstrated a further decline in the carotenoid content (Figure 1b). In addition, the ratio Chl a/b in mutant leaves was significantly higher (3.57 ± 0.04) than in *Ailsa Craig* leaves (3.29 ± 0.12), and the difference between both types of plants was maintained during the whole experimental setup (Figure 1c).

**Figure 2:**
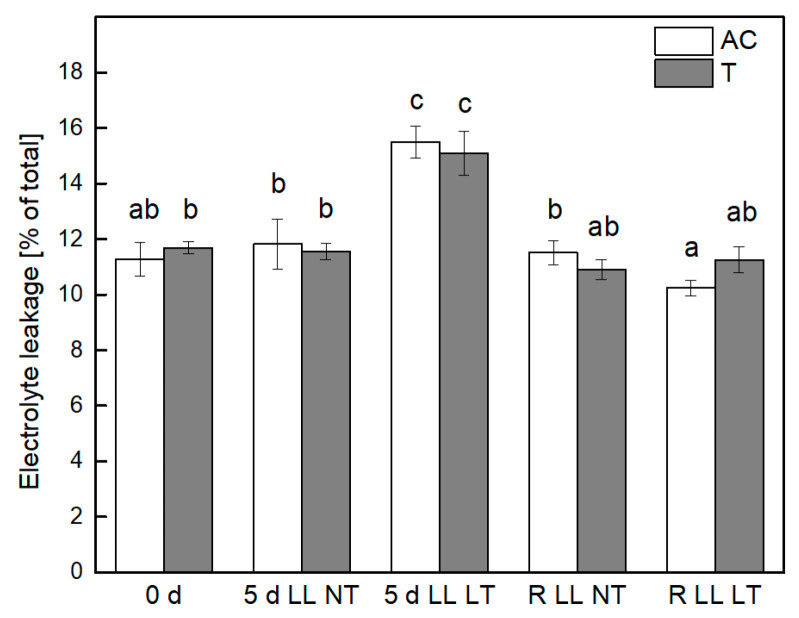
Stability of biological membranes of *Ailsa Craig* and *tangerine* leaves after exposure to low light (LL) in combination with normal (NT) or low (LT) temperature and after recovery period (R) as determined by the extent of electrolyte leakage. Mean values ± SE were calculated from two independent experiments and four parallel samples at each time point (*n* = 8). Significant differences between values at *p* < 0.05, as estimated by Fisher’s LSD test of ANOVA, were indicated with different letters.

The stability of biological membranes of tomato plants in the course of treatment by LL in combination with NT or LT was followed by the extent of electrolyte leakage from leaves of control, treated, and recovered plants (Figure 2). The values of electrolyte leakage of leaves of both types of control plants were very low, comprising 11% of the total leakage of electrolytes. A statistically significant increase, comparable for *Ailsa Craig* and *tangerine* leaves, was detected only after exposure of plants to combined treatment by LL LT, reaching 15%. The stability of biological membranes after the recovery period was improved and was comparable with that before treatment.

**Figure 3:**
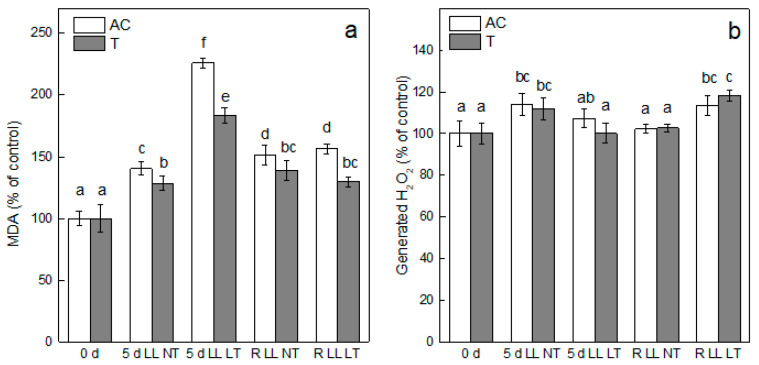
The extent of lipid peroxidation (**a**) and level of H_2_O_2_ generation (**b**) in leaves of *Ailsa Craig* and *tangerine* plants after exposure for 5 days to low light (LL) and normal (NT) or low (LT) temperature and after recovery period (R). Four parallel samples were processed at every time point of two independent experiments (*n* = 8). Every bar represents mean values ± SE. Significant differences between values at *p* < 0.05, as estimated by Fisher’s LSD test of ANOVA, were indicated with different letters. 100% for MDA—*Ailsa Craig*—27.48 ± 1.64 and for *tangerine* 34.61 ± 3.75 nmol MDA g^−1^ FW; for H_2_O_2_—*Ailsa Craig*—5.94 ± 0.35 and for *tangerine* 6.02 ± 0.31 µmol H_2_O_2_ g^−1^ FW.

The extent of oxidative stress by *Ailsa Craig* plants after LL NT and LL LT treatment was evaluated by the alterations in the level of stress markers MDA and H_2_O_2_ (Figure 3). The MDA content in tomato plants was increased by exposure to LL reaching 140% at NT and 225% at LT in comparison with control, non-treated *Ailsa Craig* plants. Treatment of *tangerine* plants led to a similar trend of elevation of MDA content, but to a lesser extent, the increase was 128% and 183% after exposure to LL NT or LL LT, respectively. Termination of treatment resulted in a significant reduction of MDA. However, for *Ailsa Craig* and *tangerine* plants the values were higher in comparison with control levels (Figure 3a). Statistically significant elevation of generated H_2_O_2_ in leaves of *Ailsa Craig* and *tangerine* tomato plants was detected only after treatment by LL NT, by 12%, in comparison with control plants (Figure 3b).

**Figure 4:**
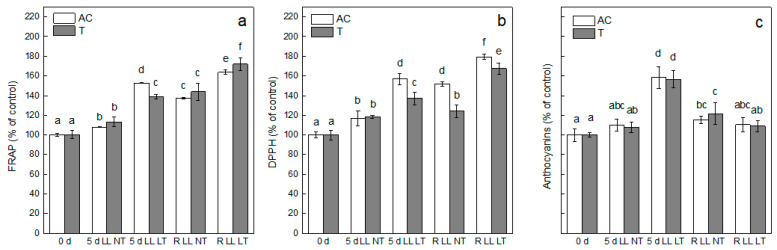
Alterations in total antioxidant (FRAP assay). (**a**) Free radical scavenging activity (DPPH assay) (**b**), and level of anthocyanins (**c**) in leaves of *Ailsa Craig* and *tangerine* plants, control (0 d), exposed for 5 days to low light (LL) and normal (NT) or low (LT) temperature and after recovery at control conditions (R). Mean values ± SE were calculated from two independent experiments with four parallel samples (*n* = 8). Significant differences between values at *p* < 0.05, as estimated by Fisher’s LSD test of ANOVA, are indicated with different letters. one hundred percent for FRAP—*Ailsa Craig*—14.07 ± 0.24 and for *tangerine* 8.94 ± 0.37 µmol F^2+^ g^−1^ FW; 100% for DPPH—*Ailsa Craig*—3.96 ± 0.13 and for *tangerine* 5.48 ± 0.26 µmol Trolox g^−1^ FW. 100% for anthocyanins—*Ailsa Craig*—0.215 ± 0.014 and for *tangerine*—0.293 ± 0.040 nmol g^−1^ FW.

The antioxidant and antiradical activities of phenolic secondary metabolites in leaves of control, treated and recovered tomato plants, *Ailsa Craig* and *tangerine*, were evaluated by FRAP and DPPH assays, respectively, and expressed as percent from the respective control (Figure 4). Application of LL treatment resulted in around a 10% increase in antioxidant activity for both types of plants (Figure 4a). More significant elevation was detected after combined treatment, LL LT, by 52%, for *Ailsa Craig* and less expressed (by 40%) for *tangerine* plants. After termination of treatment and transfer to control conditions, the antioxidant activity was further increased and values were higher in comparison with that after treatment by LL NT and LL LT. Alterations in the radical scavenging activity in leaves of *Ailsa Craig* and *tangerine* for the entire experimental setup followed the same trend—stronger increase at LL LT in comparison with LL NT treatment and enhanced values after the recovery period, valid for both types of treatment (Figure 4b).

As a response to environmental stress conditions, plants accumulate different antioxidative compounds, including anthocyanins. Tomato plants, *Ailsa Craig* and *tangerine*, responded to treatment by LL in a similar manner with respect to anthocyanin content. An elevation (by 56%) was detected after exposure to LL LT. A smaller increase (by around 10%) was detected after LL NT treatment but was not statistically significant in comparison with controls (Figure 4c). After a recovery period of 3 days, the values of anthocyanins were significantly decreased in plants previously treated by LL LT.

**Figure 5:**
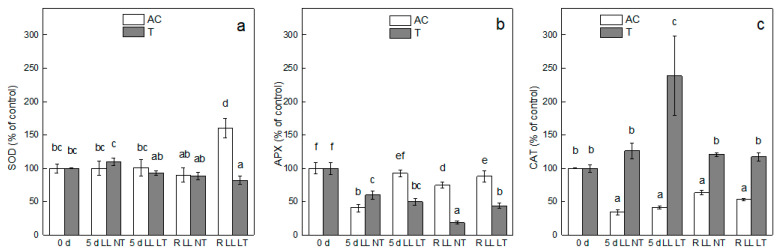
Effect of treatment by low light (LL) combined with normal (NT) or low (LT) temperature of *Ailsa Craig* and *tangerine* plants and after recovery for 3 days (R) on SOD (**a**), APX (**b**), and CAT (**c**) activity. Every bar represents mean values ± SE calculated from two independent experiments and three parallel samples at every time point (*n* = 6). The same letters within the graph indicate no significant differences assessed by Fisher’s LSD test (*p* ≤ 0.05) after performing ANOVA. 100% for SOD—*Ailsa Craig*—228.1 ± 15.9 and for *tangerine* 317.9 ± 2.1 U g^−1^ FW; 100% for APX—for *Ailsa Craig* 7474.0 ± 605.7 and for *tangerine* 9955.3 ± 894.1 U mg^−1^ FW min^−1^ and 100% for CAT—*Ailsa Craig*—898.7 ± 7.9 and for *tangerine*—312.9 ± 17.8 nmol H_2_O_2_ destroyed g^−1^ FW min^−1^.

SOD activity was not significantly affected after exposure of *Ailsa Craig* and *tangerine* to LL at both NT and LT. The most obvious increase in SOD activity was observed after 3 days of recovery of *Ailsa Craig* plants that were previously exposed for 5 days to LT LL (Figure 5a).

In contrast to SOD, APX activity significantly decreased after treatment by LL NT in *Ailsa Craig* and *tangerine* by 60% and 40%, respectively, compared to the controls (Figure 5b). After recovery at control conditions, the APX activity increased in *Ailsa Craig* but remained lower than control, whereas an additional reduction in its activity was observed after recovery of *tangerine*. The APX activity in *Ailsa Craig* was less affected when plants were exposed to LL LT compared to LL NT, whereas similar changes were observed in *tangerine* plants treated at NT and LT under LL. No significant changes in APX were detected after the recovery of plants exposed to LL LT.

CAT activity decreased in *Ailsa Craig* after treatment by LL at NT and LT (about 60%), and regardless of some enhancement, its activity remained lower than control after recovery. In contrast, exposure of *tangerine* to LL resulted in increased CAT activity, especially at LT, and it remained higher than control after recovery.

## 3. Discussion

The majority of cultivated crops originate from tropical regions and require particular for every respective crop combination of high light intensity and temperature for proper development and high yield. To meet the requirements of the growing world’s population for food such types of crops are grown under controlled conditions during fall and winter. Being a thermophilic and photophilic crop, tomato plants suffer the negative impact of receiving less light and lower ambient temperature needed for their development and fruiting during greenhouse cultivation [29]. The published data concerning the development of tomato plants at lower-than-required light illumination and lower-than-optimal temperatures are rather limited. In addition, reports on the response of tomato plants with altered carotenoid content with respect to photosynthetic performance and suffered abiotic stress, as well as antioxidative protective strategies of plants at extreme environmental conditions, are focused mainly on tomato fruits, the source of carotenoids for humans [35,36,37,40].

Here, we report on the abiotic stress suffered by tomato plants (*Solanum lycopersicum*), wild type *Ailsa Craig*, and carotenoid mutant *tangerine* after treatment for 5 days by low light illumination alone and in combination with low temperature and the applied protective strategy that enables plants to withstand the stress. The ability of tomato plants to recover at control conditions after the applied stress was monitored as well. The aim was to unravel how the altered carotenoid content of *tangerine* mutant that accumulates prolycopene instead of all-*trans*-lycopene due to defective prolycopene isomerase (CRTISO) respond to the applied double stress in comparison with *Ailsa Craig* plants. In a recent investigation, we have shown that the thylakoid membrane structure, primary photosynthetic reactions, and thylakoid membrane fluidity in *tangerine* were affected by low light and suboptimal temperature in a different manner in comparison with *Ailsa Craig*. The treatment by LL LT reduced the photochemical activity of PSII in *Ailsa Craig* to a lesser extent when compared with *tangerine*, but PSII activity in the mutant was affected negatively by LL NT and LL LT to a comparable extent. The fluidity of the *tangerine* lipid phase of the thylakoid membrane was higher in comparison with the *Aisla Craig* membranes. Data indicated that the differences between *Ailsa Craig* and *tangerine* are mainly linked to PSII photochemical activity and its antenna complexes [41].

Photosynthetic piments are indispensable components of photosynthetic organisms that are involved in absorbing and transmitting light energy. The availability of photosynthetic piments and the ratio between different pigment species is an important indicator of chloroplast development and photosynthetic performance [33]. Alterations in the pigment content of higher plants after exposure to environmental stress conditions are dependent on a number of factors, including plant species and their tolerance to the particular treatment [42]. At low light conditions, the development of chloroplasts and the synthesis of pigments in young leaves can be seriously retarded [33]. Results presented indicate that chlorophyll and carotenoid content of young *tangerine* plants under control conditions was lower (by 15% for chlorophyll and by 5% for carotenoids) in comparison with *Ailsa Craig*. However, exposure to LL or to the combined treatment LL LT decreased the piment content of *Ailsa Craig* to a more significant degree in comparison with *tangerine* plants. Both types of plants, exposed only to LL, were able to restore their pigment content during the recovery period; however, the combined treatment was more severe, and plants did not regain the pigment content of control plants. A low light-induced decline in pigment content was also detected in tobacco [43], tomato [30], and purple pakchoi (*Brassica rapa* var Chinensis) [44]. Another difference between *Ailsa Craig* and *tangerine* plants with respect to their pigment content was the higher ratio of Chl a/b in the mutant, suggesting that the light-harvesting antenna size in *tangerine* plants was smaller in comparison with *Ailsa Craig*. It had been reported that exposure to LL of low light tolerant plants resulted in higher Chl b content and lower Chl a/b ratio, thus enabling the tolerant species to absorb as much as possible sunlight [3]. During the entire experimental setup, we did not observe any statistically significant alterations in the Chl a/b ratio for both types of plants, indicating that the antenna size was not altered by treatment by LL or by LL LT, suggesting that *Ailsa Craig* and *tangerine* plants are sensitive to development at low light availability.

ROS are produced in all photosynthetic organisms, even when growing under normal conditions, and are significantly induced when plants face extreme constraints, such as high and low temperatures, high light illumination, salinity, UV, heavy metals, etc., that significantly disturb photosynthetic, biochemical and physiological functions depending on the type and duration of treatment, developmental stage, and respective genotype [1,14,15]. Development at lower than optimal light intensity can also cause oxidative stress [7,8]. The generated at the acceptor side of PSII O_2_^−^ facilitates the formation of hydroxyl radicals (OH^−^) that, due to their high redox potential and lack of scavenging enzyme, are able to attack DNA and oxidize proteins and lipids [45,46].

The level of stress-induced accumulation of the end product of peroxidation of unsaturated fatty acid chains in plant membranes, MDA, is a reliable stress indicator for the evaluation of oxidative stress under constrained conditions and damage of biological membranes [47]. The extent of lipid peroxidation in leaves of *Ailsa Craig* and *tangerine* indicated that the oxidative stress suffered by plants was much stronger expressed after exposure to the combined application of LL LT in comparison with treatment for the same period only by LL. It is worth noting that *Ailsa Craig* plants were more sensitive to both types of treatment in comparison with *tangerine* as evidenced by the level of MDA after treatments. Significant reduction of lipid peroxidation after the recovery period was detected only in plants that were subjected to combined stress; however, the values of controls were not reached, suggesting that some stress-related processes were still functioning after termination of stress treatment. It has to be mentioned that the membrane integrity, evaluated by the degree of electrolyte leakage, was disturbed in an identical manner in *Ailsa Craig* and *tangerine* only after exposure to both stress factors (LL LT) and was completely restored after recovery. It can be suggested that the higher level of MDA content is mainly due to the peroxidation of thylakoid membrane lipids that are characterized by a high amount of unsaturated fatty acids [18], while the electrolyte leakage is mainly related to the stability of cell walls.

As a response to abiotic stress, plants have developed different protective mechanisms against oxidative stress, including accumulation of protective metabolites such as proline, tocopherols, phenolic substances, etc., and overexpressing antioxidant enzymes such as SOD, APX, and CAT [14,15]. LL treatment induced increased antioxidant and antiradical activity in leaves of both types of plants that were significantly higher under combined stress (LL LT). It has to be pointed out here that the level of these activities was even higher after the termination of treatments, indicating that some residual stress-related processes remained active during the recovery period, which is in accordance with the high level of MDA after the recovery period.

Another big group of phenolic substances is anthocyanins that, in addition to performing a light screening effect under high light intensity [20,21,22], have been proven to act as more effective scavengers of generated ROS in comparison with ascorbate and tocopherol [20,21], both in vitro [48] and in vivo [20]. After treatment of *Ailsa Craig* and *tangerine* by LL, a small but not statistically significant increase of anthocyanins was detected in the plants’ leaves, which is to be expected as the main role of these metabolites is light screening under high light intensities [20,21,22]. However, the application of both stress factors, LL LT, induced a significant accumulation of anthocyanins, suggesting that under these conditions, anthocyanins are needed as scavengers of stress-induced ROS [20].

The stress-generated ROS is effectively scavenged by the cascade of superfamilies of antioxidant enzymes SOD, APX, and CAT, SOD being the primary antioxidant defense against O_2_^−^ transferring the radical to H_2_O_2_, thus providing protection against damaging oxidative action of O_2_^−^ [15,23]. The resulting H_2_O_2_ is further scavenged by APX and CAT [15,16,24,25,26]. However, it has been suggested that LL-induced antioxidant enzyme activities are largely dependent on the plant species with respect to its ability to tolerate the respective type of stress. Low light-tolerant species can maintain high antioxidative enzyme activities that provide significant protection against the negative effects of stress-generated ROS on plant physiology and performance, while low light-sensitive varieties fail to provide sufficient antioxidant enzyme protection [3]. The induction of antioxidant enzymes at the application of LL is not unequivocal. An increase in SOD activity and MDA content was detected in the Jinfen 5 variety of tomato (*Solanum lycopersicum*), while a reduction in CAT activity was detected under combined treatment for 10 days by LT and weak light. However, the exposure was longer and at much lower light intensities in comparison with our set up [30]. A decrease in SOD and APX activity was also detected in three tomato genotypes after exposure to LL in combination with LT [49], as well as a decline in APX in purple pakchoi (*Brassica rapa* var. Chinensis) under LL [44]. Under our experimental conditions, we did not observe an increase in antioxidant enzyme activities of SOD, APX, and CAT after exposure to LL in combination with NT or LT, implying the sensitivity of *Ailsa Craig* and *tangerine* plants to LL. An elevation of CAT activity was detected only in *tangerine* at LL application that was stronger expressed when LL was combined with LT. It can be supposed that the lower sensitivity of *tangerine* plants to the treatment, as evidenced by MDA content, can be due to the antioxidant support of the CAT enzyme.

## 4. Materials and Methods

### 4.1. Plant Material

In this investigation, we used tomato plants of the *tangerine* mutant (LA3183) and its nearly isogenic wild type “*Ailsa Craig*” (+/+) (LA2838A) that were provided by the Tomato Genetics Resource Center, Davis, CA, USA. Tomato plants were grown as described by Velitchkova et al. [41] and Gerganova et al. [50]. Seeds of *Ailsa Craig* and *tangerine* were initially placed on moist filter paper for 48 h at room temperature and further transferred to pots filled with perlite-containing soil and kept at 4 °C for 4 days. Afterward, the plants were grown in growth chambers (*Fytoscope FS130, Photon Systems Instruments*, Drásov, Czech Republic) for about 22 days. The photocycle was 16 h/8 h (day/night), light intensity was 250 µmol photons m^−2^ s^−1^ (PFD) (NL), temperature 24/22 °C (day/night) (NT), and relative humidity 75%. After the emergence of the third leaf, the plants were exposed for 5 days at low light intensity (125 µmol photons m^−2^ s^−1^) (LL) in combination with normal (NT) (24/22 °C) or low temperature (LT) (15/10 °C). After the treatment, plants were transferred to control conditions (NL NT) for 3 days of recovery (R). Samples were taken from different leaves at the beginning of each experiment (0 d) after 5 days of treatment (5 d LL NT, 5 d LL LT) and after 3 days of recovery (R LL NT, R LL LT). Two independent experiments were performed, and 4 parallel samples were evaluated at every time point. The conditions that plants were grown before the start of treatment, light illumination, and temperature, were indicated as control conditions throughout the text.

### 4.2. Determination of Photosynthetic Pigment Content

Pieces of different leaves from control, treated, and recovered plants (40 mg for each sample) of *Ailsa Craig* and *tangerine* mutant were ground with a mortar and pestle with ice-cold 80% (*v*/*v*) acetone in dim light [50]. The homogenate was centrifuged in sealed tubes at 4500× *g* for 15 min at 4 °C. The clear extract was used to spectrophotometrically (*UV-VIS Specord 210 Plus*, Analytic Jena, Jena, Germany) determine the total content of chlorophyll (Chl (a+b)) and carotenoids (Car) using the coefficients and formulas of Lichtenthaler (1987) [51]. Mean values ± SE were calculated from two independent experiments and 4 parallel samples at every time point and expressed as mg pigment g^−1^ FM.

### 4.3. Electrolyte Leakage

Pieces of leaves from control, treated, and recovered plants (150 mg) were transferred to 15 mL double distilled water. The samples were kept at room temperature and gentle shaking for 24 h. The conductivity of the floating solution was measured with a conductivity meter (*HI5321*, Hanna Instruments, Smithfield, RI, USA). The maximum leakage of the plant tissue was determined after boiling the leaf material for 15 min at 100 °C. The extent of leakage was presented as a percentage of the maximum leakage.

### 4.4. Lipid Peroxidation and H_2_O_2_ Content

The extent of lipid peroxidation in leaves of *Ailsa Craig* and *tangerine* plants was determined by the malondialdehyde (MDA) content following the thiobarbituric acid method (TBA) [52]. Pieces of different leaves (100 mg) were homogenized in 3 mL 0.1% (*w*/*v*) trichloroacetic acid (TCA) at 4 °C. After centrifugation at 4500× *g* for 15 min at 4 °C 1 mL of the clear extract was mixed with the same volume 20% TCA containing 0.5% TBA and was boiled in a water bath for 25 min. After cooling, the absorbance at 532 and 600 nm was recorded (*UV–VIS Specord 210 Plus*). The absorbance at 600 nm was read to correct for unspecific turbidity. The amount of formed TBA-reactive metabolites (aldehydes, mainly MDA and endoperoxides) was calculated using the extinction coefficient of 155 mM^−1^ cm^−1^. The results were expressed on a fresh weight basis [nmol MDA g^−1^ FW].

The supernatant, after centrifugation of homogenized leaves material with 0.1% TCA, was used for the determination of generated H_2_O_2_ content. To 0.5 mL of the supernatant was added 0.5 mL K phosphate buffer (pH 7.0) and 1 mL 1 M KI. The mixture was kept in the dark for 2 h and was periodically vortex-mixed. The absorbance at 390 nm was spectrophotometrically determined (*UV–VIS Specord 210 Plus*). The amount of H_2_O_2_ was determined using a standard curve of known H_2_O_2_ concentrations [53]. Results were expressed on a fresh weight basis [µmol H_2_O_2_ g^−1^ FW].

### 4.5. Determination of Anthocyanin Content

Pieces of leaves from different *Ailsa Craig* and *tangerine* plants—control, treated, and recovered plants (50 mg) were homogenized in a 6 mL medium containing ethanol/HCl/H_2_O (79/1/20, *v*/*v*/*v*) and centrifuged at 10,000× *g* for 15 min at 4 °C. The clear extract was used to determine anthocyanin content. The absorbance at 535 and 653 nm was recorded. Anthocyanin content was calculated by the formula A535 − 0.24 × A653 [54] and molar extinction coefficient 33,000 M^−1^ cm^−1^ [55]. Results were presented as [µmol g^−1^ FW]. At every time point of each experiment four parallel samples were processed.

### 4.6. Antioxidant and Free Radical Scavenging Activity of Phenolic Metabolites

The antioxidant activity and free radical scavenging activity of phenolic metabolites in *Ailsa Craig* and *tangerine* plants were accessed as described by Doncheva et al. [56]. Leaf material (500 mg) from control, treated, and recovered plants were ground at 4 °C in the dark with 80% ethanol. The resulting homogenate was centrifuged at 12,000× *g* at 4 °C for 30 min. The supernatant was used for the determination of antioxidant and free radical scavenging activities.

The total antioxidant activity was determined following the ferric-reducing antioxidant power (FRAP) assay [57]. The reduction of ferric ions (Fe^3+^) to ferrous ions (Fe^2+^) is performed by the available bioactive substances (antioxidants). To 0.05 mL of the ethanol extract were added 1.5 mL of freshly prepared FRAP reagent and 0.15 mL of distilled water. The reaction was carried out at room temperature. A blue color developed after 15 min, and the absorbance was recorded at 593 nm. FRAP solution served as the blank. The antioxidant activity of the samples was determined using a standard curve with known concentrations of FeSO_4_·7H_2_O.

The free radical scavenging activity was evaluated as described by Brand-Williams et al. [58]. DPPH (1,1-Diphenyl-2-picrylhydrazyl) was applied as the free radical source. The discoloration of DPPH solution is dependent on the available in the sample radical species or antioxidants. Freshly prepared DPPH reagent (1.99 mL) was mixed with 0.01 mL of the ethanol extract. The scavenging process was conducted in the dark at ambient temperature for 30 min. In parallel, one test tube with 2 mL of pure DPPH solution (without ethanol extract) was placed for comparison of the degree of discoloration. The absorbance at 515 nm was spectrometrically recorded. Methanol was used as the blank. A standard curve with known Trolox concentrations was used to calculate the free radical scavenging capacity.

### 4.7. Enzyme Activity Assays

Pieces of leaves (200 mg) from different plants of control, treated, and recovered *Ailsa Craig* and *tangerine* plants were collected and immediately frozen in liquid nitrogen and kept at –80 °C for further evaluation. Leaf material was ground at 4 °C with 1.2 mL 50 mM K phosphate buffer (pH 7.8) supplemented with 0.1 mM EDTA. The homogenate was centrifuged at 15,000× *g* for 20 min at 4 °C. The pellet was resuspended in 0.8 mL of the same buffer and centrifuged again. Both supernatants were combined and used to evaluate the enzyme activities of SOD, APX, and CAT [59].

The activity of superoxide dismutase (SOD; EC 1.15.1.1) was determined according to [60] by monitoring the superoxide radical-induced nitro blue tetrazolium (NBT) in the presence of riboflavin reduction at 560 nm (*UV–VIS Specord 210 Plus*). The reaction mixture contained 50 mM K phosphate buffer (pH 7.8), 0.1% Triton X-100, 0.1 mM EDTA, 0.06 mM NBT, 10 mM methionine, 2 µM riboflavin, and enzyme extract. One unit (U) of SOD was the amount of enzyme that caused 50% inhibition of NBT reduction in light and expressed as [U g^−1^ FW].

The activity of ascorbate peroxidase (L-Ascorbate:H_2_O_2_ oxidoreductase) (APX; EC 1.11.1.11) was assayed by monitoring the rate of hydrogen peroxide-dependent ascorbate oxidation at 290 nm by *UV–VIS Specord 210 Plus* [61]. The reaction medium contained 25 mM K phosphate buffer (pH 7.0), 0.5 mM ascorbate, 2 mM H_2_O_2_, 0.1 mM EDTA, and enzyme extract. One U of APX was determined as the decrease of absorbance at 290 nm with 0.001 and was expressed as [U g^−1^ FW min^−1^].

The catalase (H_2_O_2_: H_2_O_2_ oxidoreductase) (CAT; EC 1. 11.1. 6) activity was assayed according to [62] by the time-dependent decline in absorbance at 240 nm (*UV–VIS Specord 210 Plus*) related to the extent of H_2_O_2_ decomposition at 25 °C in a reaction mixture containing 25 mM K phosphate buffer (pH 7.0), 10 mM H_2_O_2_ and enzyme extract. The activity of CAT was determined by the amount of degraded H_2_O_2_ for 1 min and expressed as [nmol H_2_O_2_ g^−1^ FW min^−1^].

### 4.8. Statistics

Results in all figures were presented as mean values ± SE, calculated from two independent experiments with four parallel samples at every time point. Mean values were statistically compared by the Fisher’s least significant difference (LSD) test at *p* ≤ 0.05 following analysis of variance by ANOVA. All values in every panel were simultaneously statistically compared. Statistically different values were indicated by different letters. A statistical software package (StatGraphics Plus, version 5.1 for Windows, The Plains, VA, USA) was used.

## 5. Conclusions

Taken together, the results about membrane integrity and lipid peroxidation in the leaves of wild-type *Ailsa Craig* and *tangerine*, a mutant that accumulates prolycopene instead of all-*trans*-lycopene, indicated that the combined action of low light and low temperature was causing much stronger oxidative stress when compared to exposure only to low light. In addition, the high MDA content and high levels of antioxidant and antiradical activities after the recovery period indicated that after 5 days of treatment by low light and low light in the presence of low temperature, there were still active stress-related processes. Protection against generated ROS was provided by increased levels of phenolic compounds, including anthocyanins. The sensitivity of the *tangerine* mutant was less expressed than that of *Ailsa Craig*. Significant protection against oxidative stress in *tangerine* was provided by the high activity of CAT antioxidant enzyme under conditions of low light, combined with control or low temperature.

## Data Availability

Data are contained within the article.
